# Ischemic stroke and disseminated tuberculosis in a child living with human immunodeficiency virus: a case report and review of the literature

**DOI:** 10.1186/s13256-019-1990-2

**Published:** 2019-02-20

**Authors:** Tinsae Alemayehu, Ayalew Moges, Ayal Mekuanint

**Affiliations:** Department of Pediatrics and Child Health, College of Health Sciences, Addis Ababa, University, Addis Ababa, Ethiopia

**Keywords:** Acute ischemic stroke, HIV, Tuberculosis, Extrapulmonary, Ethiopia

## Abstract

**Background:**

Cerebrovascular accidents are rare in children. Rates of stroke in children with human immunodeficiency virus infection are higher than in the uninfected population.

**Case presentation:**

We report the case of a 19-month-old Ethiopian boy who presented with a left-sided body weakness of sudden onset. He was also diagnosed as having human immunodeficiency virus infection. Laboratory tests showed an iron deficiency anemia and imaging revealed tuberculosis of his lungs, spleen, and abdominal lymph nodes as well as an acute ischemic stroke of the right middle cerebral artery region. His symptoms improved after anti-tuberculosis drugs, antiretroviral treatment, and iron supplementation were initiated.

**Conclusions:**

Extrapulmonary tuberculosis should be considered a cause of sudden focal neurologic deficits in children with human immunodeficiency virus infection residing in endemic countries.

## Background

Childhood stroke, ischemic or hemorrhagic, is rarely diagnosed. Ischemic stroke is a result of neurologic insult due to impaired perfusion [1]. Predisposing factors are broadly categorized into cardioembolic, hematological, vasculitis related, neoplastic, drug related, and cryptogenic. Common underlying disorders in developing countries may include: iron deficiency anemia; infections such as human immunodeficiency virus (HIV), herpes simplex virus, syphilis, tuberculosis (TB), and invasive mycoses; infective endocarditis; and disseminated intravascular coagulation (DIC) [2]. HIV infection can complicate cerebrovascular diseases in various ways. Opportunistic infections, HIV-associated vasculopathy, HIV myocarditis, and coagulation defects like protein S deficiency and anti-phospholipid antibodies are reported to be associated events [3]. Reports on the co-occurrence of HIV infection and cerebrovascular diseases are sparse. Incidence rates among children with perinatally acquired HIV are around 3.3 cases per 10,000 person-years, higher than their uninfected counterparts [4]. Fulmer *et al.* placed the incidence of radiologic evidence for stroke among children with HIV infection at 1% [5]. A retrospective review of clinical and neuroimaging features of pediatric HIV-1 encephalopathy in Addis Ababa, Ethiopia, found a single child with ischemic stroke among the 22 studied [6].

We present the case of a 19-month-old boy, newly diagnosed with HIV infection, with acute ischemic stroke and disseminated TB.

## Case presentation

A 19-month-old Ethiopian baby boy from Addis Ababa, Ethiopia, presented with a left-sided body weakness of 4 days’ duration to Tikur Anbessa Specialized Hospital. The weakness of his left upper and lower extremities was noted by his mother upon awakening from sleep. He also had a low grade intermittent fever and weight loss (not quantified) for the preceding 1 month. His mother had symptoms of cough, sweating, and weight loss for the past 3 months for which she did not seek medical attention.

He was born at term to a primiparous woman, diagnosed as having HIV infection since her second month of pregnancy. Antiretroviral treatment (ART) was initiated along with diagnosis and she delivered via caesarean section. She opted for exclusive breast feeding. The newborn was given nevirapine prophylaxis immediately after delivery but both the mother’s and neonate’s ART were discontinued on the third day of life due to poor social support for the family. The child did not receive any further care for exposure to HIV infection. He had received all the vaccines of the national immunization schedule. His developmental milestones were optimal.

On physical examination, his vital signs were within normal limits. He was stunted with height measuring 71 cm (less than 5th centile for age). He had pale conjunctivae with 1.5 by 1 cm right and left axillary lymphadenopathies. He was fully conscious. A neurologic examination revealed left-sided hypertonia, hyper-reflexia, and weakness (left upper extremity 0/5 and left lower extremity 3/5).

A complete blood count showed white blood cell (WBC) of 5700/mm^3^ with 64% neutrophils and 23% lymphocytes. His hemoglobin was 7.6 gm/dl, mean corpuscular volume (MCV) 66.1 fl, and platelets 261,000/mm^3^. Erythrocyte sedimentation rate (ESR) was 107 mm/hour and HIV serology test was reactive. Baseline tests showed a CD4 count of 320/mm^3^, CD4 percentage of 14%, and a viral load of 690,000 copies/ml. Serum Venereal Disease Research Laboratory (VDRL) test was non-reactive. Cerebrospinal fluid (CSF) analysis had no cells, with normal CSF glucose and protein. CSF VDRL was non-reactive. Organ function tests and lipid profiles were within normal limits. Coagulation profile tests showed a prothrombin time (PT) of 14.4 seconds, activated partial thromboplastin time (PTT) of 23.1 seconds, and international normalized ratio (INR) of 1.13. Testing for serum protein C and protein S levels was not possible in our hospital.

**Fig. 1:**
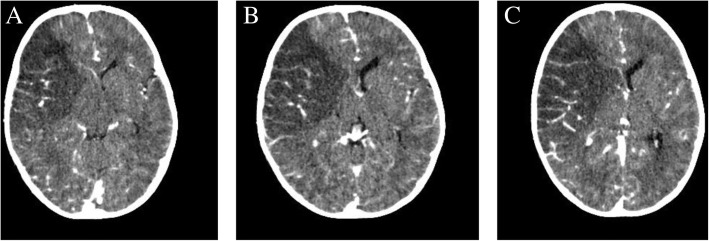
**a–c**: Computed tomography of the brain showing a cerebral and basal ganglia hypodensity with loss of gray white matter differentiation in the M1 segment of the right middle cerebral artery territory (acute ischemic infarct) and effacement of the right lateral ventricle with a leftwards midline shift

A chest X-ray revealed bilateral, mainly central, nodular opacities. Echocardiography was normal. Multiple hypoechoic splenic lesions and periportal lymphadenopathy were evident on an abdominal ultrasound. A computed tomography (CT) scan of his brain (Fig. 1 a–c) showed right temporoparietal M1 segment of middle cerebral artery territory acute ischemic infarct.

A CT angiography of his brain confirmed the above findings along with hemorrhagic transformation.

A diagnoses list of stunted, disseminated TB (lung, spleen, abdominal lymph nodes, and brain), iron deficiency anemia, left-sided hemiparesis due to ischemic stroke possibly due to tuberculous vasculitis, and a newly diagnosed HIV infection was made. Co-trimoxazole prophylaxis, anti-TB treatment (isoniazid, rifampicin, pyrazinamide, and ethambutol) along with steroids and pyridoxine, aspirin, and iron supplementation were initiated as well as physiotherapy. Within 2 weeks of starting treatment, ART with abacavir, lamivudine and lopinavir/ritonavir was started.

He has currently completed his anti-TB treatment (2 months of isoniazid, rifampicin, pyrazinamide, and ethambutol and 10 months of isoniazid and rifampicin) as well as being on ART. His examination shows normal tone and reflexes with his left upper extremity having a power of 3/5 but his left lower extremity having a power of 5/5.

## Discussion and conclusions

Improved diagnostic tools have led to more reports of cerebrovascular accidents in people with HIV infection in sub-Saharan Africa. A retrospective study conducted in Tikur Anbessa Hospital, Ethiopia, found that stroke accounted for 2.3% of 347 admissions with HIV infections and neurologic manifestations [7].

Presentations can range from asymptomatic episodes to acute focal neurologic deficits including focal abnormal body movements to headache and speech difficulties [8]. The circle of Willis and basal locations (basal ganglia and thalamus) are reported to be commonly affected [9]. A complete blood count, organ function tests, ESR, lipid profile, CSF analysis, and coagulation screening are important screening tests. Imaging, such as CT, magnetic resonance imaging (MRI), and magnetic resonance angiography (MRA), may reveal basal, focal cerebral or internal capsule infarction, or stenosis, occlusion or aneurysmal dilatation of major vessels, especially middle cerebral, distal internal carotid, and proximal anterior cerebral arteries [2, 10].

Aneurysms and cerebral vasculitis leading to stroke can be related to the HIV infection itself or opportunistic infections like TB. Tuberculous vasculitis, in particular, may cause inflammatory vasculopathy (narrowing of vessels and multiple infarctions) especially around the circle of Willis due to basal exudates [11].

Our patient was newly diagnosed as having HIV infection (he was not on highly active antiretroviral therapy or any drugs known to be risks to develop stroke) with an acute ischemic stroke. Disseminated TB involving his lung, spleen, abdominal lymph nodes, and brain was also diagnosed. He had no evidence for tuberculous meningitis. His presentation is probably related to vasculopathy due to TB compounded by iron deficiency anemia. Determining serum protein C and S would have completed our set of tests for his cerebrovascular accident but there was no evidence of other etiologies (cardiac disorders, DIC, neoplasms, other opportunistic infections, and so on) barring TB.

Correlations between cerebrovascular accidents and disseminated TB in the absence of TB meningitis are rarely reported. We describe a child with HIV infection with acute ischemic stroke, disseminated TB, and iron deficiency anemia. We underline the need to screen for extrapulmonary TB in children with HIV infection presenting with sudden focal neurologic deficits in countries endemic for the disease.

## Abbreviations

ART: Antiretroviral treatment
CSF: Cerebrospinal fluid
CT: Computed tomography
DIC: Disseminated intravascular coagulation
ESR: Erythrocyte sedimentation rate
HIV: Human immunodeficiency virus
INR: International normalized ratio
MCV: Mean corpuscular volume
MRA: Magnetic resonance angiography
MRI: Magnetic resonance imaging
PT: Prothrombin time
PTT: Activated partial thromboplastin time
TB: Tuberculosis
VDRL: Venereal Disease Research Laboratory
WBC: White blood cell

## References

[1] Tsze DS, Valente JH. Pediatric Stroke: A Review. Emerg Med Intl. 2011;734506:1–10. DOI 10.11 55 10.1155/2011/734506 PMC3255104 22254140

[2] Hammond CK, Eley B, Wieselthaler N, Ndondo A, Wilmshurst J. Cerebrovascular disease in children with HIV-1 infection. Dev Med Child Neu. 2016;58:452–60. 10.1111/dmcn.13080. 26890389

[3] Benjamin LA, Bryer A, Emsley CA, Khoo S, Solomon T, Connor MD. HIV infection and stroke: current perspectives and future directions. Lancet Neurol. 2012;11(10):878–90. 10.1016/S1474-4422(12)70205-3 PMC3460367 22995692

[4] Schieffelin JS, Williams PL, Djokic D, *et al.* Central nervous vasculopathy in HIV-infected children enrolled in the Pediatric AIDS Clinical Trials Group 219/219C Study. J Ped Infect Dis Soc. 2013;2(1):50–6. 10.1093/jpids/pis095 PMC8900258 26619442

[5] Fulmer BB, Dillard SC, Musulman EM, *et al.* Two cases of cerebral aneurysms in HIV+ children. Pediatr Neurosurg. 1998;28(3):1–4. 10.1159/000028615 9693327

[6] G Mariam A, Assefa G. Clinical and neuroimaging profile of HIV-1 encephalopathy in infancy and childhood in a sub-Saharan African country. Ethiop Med J. 2012;50(3):337–47. 23930479

[7] Berhe T, Melkamu Y, Amare A. The pattern and predictors of mortality of HIV/AIDS patients with neurologic manifestation in Ethiopia: a retrospective study. AIDS Res Ther. 2012;9:11. 1–7 10.1186/1742-6405-9-11 PMC3348055 22490062

[8] Ranzan J, Rotta NT. Ischemic stroke in children. Arq Neuropsiquiatr. 2004;62(3-A):618–25. 10.1590/s0004-282x2004000400010 15334219

[9] Karabsi-Afshar R, Izadi M. HIV and stroke events: a systemic review and meta-analysis. Intl J Med Rev. 2014;1(1):1–10.

[10] Patsalides AD, Wood LV, Atac GK, Sandifer E, Butman JA, Patronas NJ. Cerebrovascular disease in HIV-infected pediatric patients: neuroimaging findings. Am J Roentgenol. 2002;179(4):999–1003. 10.2214/ajr.179.4.1790999 12239054

[11] Wang Y, Li Q, Zhen X, Liu Y, Wu Q. Immunologic cerebral vasculitis and Extrapulmonary Tuberculosis: An Uncommon Association. J Clin Diagn Res. 2015;9(9):3–5. 10.7860/JCDR/2015/13885.6497 PMC4606267 26500938

